# Homœopathy in Our Colleges

**Published:** 1887-03

**Authors:** G. W. Sherbino

**Affiliations:** Abilene, Texas


### "HOMŒOPATHY IN OUR COLLEGES."

G. W. Sherbino, Abilene, Texas.

Are the colleges doing their duty? They are all named, but do they teach according to that *name?* I think not. Some of the clinical instructions are good, and some are bad and smattered with the old-school teaching. This should never be allowed in a college called homœopathic. Those who do depart from it are hypocrites and pretenders. I want to put my protest against such departures as being wrong in teaching allopathy to our students.

For instance, a student is just commencing to progress in the light of medical knowledge of to-day.

We will take first the chair of Gynæcology. The professor has a woman brought into the clinic for examination to teach the students the art of diagnosis and treatment; the case is examined so they can all learn and know how to manipulate, use the instruments with skill and precision. The case shows enlargement and congestion; the subjective symptoms are not looked after at all, nor are they desired, as this case must be one of surgery, and we will cut the anterior lip and bleed the cervix and relieve the engorgement. About one-half teacup of blood is caught. In one week she reports for another treatment. She gets it this time in the form of strong, fuming Nitric acid introduced into the "*endometrum.*" Any one in the class could see the fumes. She came back the third time, and he said he never saw such an inactive, stubborn case. He cut the anterior lip again. This time he gave Actea rac. without any indication whatever. This professor had a fine practice, was a gentleman, a fine operator, and possessed suavity and affability in a marked degree.

Second case was seen at another professor's clinic. "One case I remember very distinctly, was examined with the much abused speculum—another case of congestion." *He told the class he would give her Aconite.*

The professor of obstetrics, who was a very fine teacher, gave his class instructions in "Post partum" hemorrhage, to inject above the pubis "fluid extract of Ergot," and for constipation "Sulphur and molasses." He thought beer also good, as the Germans, who drink beer, never get constipated.* It was very seldom that he gave us therapeutical indications for parturient patients. He had tried the high potencies, two hundredth, and they were a total failure. Any man that would say that there were any curing properties in them was a —— liar.

Are the boys getting such Homœopathy as this this winter? Is it any wonder that so many depart from the "*master's*" teaching with this kind of allopathic treatment? Let us see to it that our students are taught Homœopathy, and to see that they are sent to colleges where they will get the true principles of our healing art in its "simplicity and purity."

Let our united voice be raised in condemnation of this pernicious and barbarous treatment. And may it be buried in the depth of oblivion, and sunk down deeper and deeper out of sight, only to be raised at the last day, with the maimed and the cripples, those who suffered the pangs of death and were made martyrs and lost their lives in this BLIND and inhuman treatment.

Don't send students where they will be spoiled by wrong teaching. "Watch them and cherish them as the apple of your eye." There are many, very many instances, where the students received

---

*Aloes in the beer.

good and wholesome instruction. They were as innocent as a young child who had never seen any other than good, sound Homœopathy. But they were sent away to college. They came back to teach the "*old doctor*" who had given them three years of instruction "all for love." He saw those same children come home filled full of such teaching as I have related, and I can tell you it's no dream or imagination, but truth. They have had all of the good lessons physicked out of them. They are of age, and they set up with tinctures, *Warner's parvules and granules and pink* medicines.

How important it is that every one of our colleges should have at least one professor who has the manliness to give our students the teaching of Hahnemann, and instruct them in the knowledge of our law, and build their young minds up in the most holy faith of *Similia similibus curantur.* This branch of teaching* is far away from the sound doctrines of Homœopathy. The most important branches are to know how to heal the sick; "*individualizing*" the single remedy and the minimum dose of the dynamized drug. How many chairs in our colleges are giving the students this kind of teaching! A student that is cast out upon the world to battle with diseases with no better training than some of them have, as far as the principles of Homœopathy are concerned, is like a mariner upon the broad ocean without a "*compass*" *to guide him among the bewildering and almost innumerable symptoms in the treatment of disease.*

*As the twig is bent, so the tree inclines.*

The environments and all conditions, circumstances, and surroundings have a wonderful influence on all organisms, especially man.

As the students are taught by their preceptors and professors, so do they partake of their teachings, and as a natural consequence follow in their footsteps. I have observed always that doctors who use absolutely tinctures and low potencies and crude doses have little regard for our system of medicine, further than to use it as a cloak, as an advertisement; "who put their cards in the paper as homœopathists," and when they make their visits they will tell their sick patients they understand both systems of medicine and will give them "*either, or both mixed.*" Such are practicing *all for money*, and they know about as much of Homœopathy as I do of the navigation of the Hudson River.

* The teaching of these homœopathic professors—so-called.

